# Gestational Diabetes Mellitus Is Associated with Age-Specific Alterations in Markers of Adiposity in Offspring: A Narrative Review

**DOI:** 10.3390/ijerph17093187

**Published:** 2020-05-04

**Authors:** Shila Shafaeizadeh, Louise Harvey, Marieke Abrahamse-Berkeveld, Leilani Muhardi, Eline M. van der Beek

**Affiliations:** 1 Nutricia Research, Uppsalalaan 12, 3584 CT Utrecht, The Netherlands; louise.harvey@danone.com (L.H.); marieke.abrahamse@danone.com (M.A.-B.); or e.m.van.der.beek@umcg.nl (E.M.v.d.B.); 2 Danone Early Life Nutrition, Cyber 2 Tower, 15th Floor, Jl. HR. Rasuna Said #X–5 No. 13, South Jakarta 12950, Indonesia; lestarina@hotmail.com; 3 Department of Pediatrics, University Medical Centre Groningen, CA84, Room Y2.115, Hanzeplein 1, 9713 GZ Groningen, The Netherlands

**Keywords:** gestational diabetes, offspring, longitudinal growth, adiposity, skin fold thickness, body mass index, weight gain, diet, maternal nutrition, breastfeeding

## Abstract

Maternal hyperglycemia alters an offspring’s metabolic health outcomes, as demonstrated by the increased risk for obesity, impaired glucose handling and diabetes from early childhood onwards. Infant growth patterns are associated with childhood adiposity and metabolic health outcomes and, as such, can be used as potential markers to detect suboptimal metabolic development at an early age. Hence, we aimed to assess whether gestational diabetes mellitus (GDM) has an impact on offspring growth trajectories. Outcomes included weight gain (WG), body mass index (BMI), and skin fold thickness (SFT) measured at least at two time points from birth to later childhood. In addition, we explored the role of early life pre- and post-natal nutritional modifiable factors on longitudinal growth in infants of mother with GDM (GDM–F1). Despite the large heterogeneity of the studies, we can still conclude that GDM seems to be associated with altered growth outcomes in the offspring. More specifically, these alterations in growth outcomes seem to be rather time-specific. Increased SFT were reported particularly at birth, with limited information on reporting SFT between 2–5 y, and increased adiposity, measured via SFT and BMI, appeared mainly in later childhood (5–10 y). Studies evaluating longitudinal growth outcomes suggested a potential role of early life nutritional modifiable factors including maternal nutrition and breastfeeding. These may impact the cycle of adverse metabolic health by attenuating growth outcome alterations among GDM–F1. Conclusions: Timely diagnoses of growth deviations in infancy are crucial for early identification of GDM–F1 who are at risk for childhood overweight and metabolic disease development.

## 1. Introduction

From conception to birth, specific changes in maternal metabolism ensure an adequate supply of nutrients to the fetus. Insulin resistance increases gradually under the influence of placental hormones, increasing the requirements for maternal insulin to maintain adequate glycemic control during pregnancy [1,2]. Glucose metabolism adapts adequately to the changing needs during pregnancy. However, in the case of insufficient pancreatic capacity, gestational diabetes mellitus (GDM) may develop [3]. GDM currently affects 1 out of 7 births worldwide [1]. Risk factors predisposing to GDM include excessive pre-pregnancy adiposity, advanced maternal age, a family history of type 2 diabetes mellitus (T2DM), a history of GDM, not exposed to breastfeeding in a previous pregnancy, a previous large or macrosomic baby, short maternal stature, polycystic ovary disease, previous stillbirth, high blood pressure during pregnancy, multiparity, as well as an unhealthy diet and sedentary lifestyle [4,5]. The median prevalence of GDM ranges from 12.9% in the Middle East followed by around 11% in Southeast Asia, Western Pacific, and South and Central America to 5.8% in regions in Europe [6]. It must be noted that aside from the different ethnic backgrounds, the substantial variation in the prevalence of GDM in each country is also influenced by the different screening and diagnostic criteria used [6].

Although GDM most often resolves post-partum, it can have immediate and long-term adverse health outcomes for both mother and offspring. Women with GDM are at risk of developing hypertension disorders during pregnancy (including pre-eclampsia) and are more prone to develop T2DM (50% affected within 10 y of a GDM pregnancy) [1,7]. The excess supply of nutrients can lead to fetal hyperinsulinemia and increased fetal growth, resulting in a higher prevalence of macrosomia, shoulder dystocia, respiratory distress, hypoglycemia, polycythemia, hyperbilirubinemia, and caesarean delivery of the new-born infant [8]. Moreover, hyperglycemia in the intrauterine environment has an impact on the offspring’s metabolism, as evidenced by their increased risk for obesity, impaired glucose handling, metabolic syndrome, and T2DM in later life [9]. Thus, GDM is a condition that fuels a viscious circle of predisposition to metabolic disorders across generations.

Fetal and infant growth trajectories play a pivotal role in the development of childhood obesity and associated metabolic outcomes. A deeper understanding of the impact of GDM on offspring growth and body composition development is essential to identify critical windows for metabolic development and/or the development of suitable intervention strategies. Moreover, understanding the impact of early life modifiable factors on adiposity alterations in GDM offspring is of importance; especially important is evaluating the impact of perinatal maternal diet (pregnancy and breastfeeding) and infant feeding pattern on offspring obesity risk throughout life [10].

Longitudinal growth outcomes of offspring born from women with GDM (GDM–F1) are poorly described, particularly in relation to their use as potential early markers of metabolic disorders. Most of the available studies either focus solely on birth outcomes or provide a cross-sectional analysis of childhood growth outcomes and lack a clear longitudinal and follow up overview of ages at which growth alterations may become evident. Formulation of preventive strategies for the development of childhood obesity in this particular population require evaluation of longitudinal growth measurements [11]. Thus, the aim of this narrative review was to determine whether GDM–F1 offspring show time-specific alterations in growth outcomes such as weight gain (WG), body mass index (BMI), and adiposity (as measured by skin fold thickness (SFT)) during infancy, and childhood. As such, we primarily reviewed studies in which one of those parameters was measured at a minimum of two different time points. In addition, we also focused on the relationship between maternal nutrition and feeding patterns (as two nutritional modifiable factors) and longitudinal growth outcomes among GDM–F1.

## 2. Review of the Evidence

We observed a large heterogeneity in the reviewed studies reporting longitudinal growth data in GDM offspring. Apart from differences in reported growth outcome parameters, e.g., using standard deviation (SD) scores, percentiles or absolute values, a large variety of assessment ages and number of data collection timepoints were apparent across studies. Moreover, different reference groups were used, such as women with normal glucose tolerance (NGT–F1) [12,13,14,15,16,17,18,19,20,21,22,23]; women with pre-gestational diabetic (Type 1 and Type 2) [16,24,25], women with milder levels of glucose intolerance [21,26], World Health Organization (WHO) standards or country specific reference data [27]. Despite this large heterogeneity, we aimed to summarize the data in a narrative review that aims to provide insight into alterations in growth outcomes of GDM–F1 as potential early predictive markers for childhood overweight risk. We provide details of our search strategy in the Supplementary Methods.

### 2.1. Longitudinal Growth Outcomes in Offspring Born to GDM vs. Control Group

Differences in WG, BMI and SFT between GDM–F1 and different control groups were reported over a wide age range (Table 1), e.g., from 0–5 y; 5–10 y and ≥ 10 y. More information is provided in Supplementary Tables S1 and S2.

### 2.2. Pattern of Weight Gain among GDM–F1

Weight gain in early infancy is more rapid than any other post-natal period of life and rapid weight gain in infancy is a well-established marker of possible alterations in metabolic health in later life [28].

Interestingly, during the first year of life, both increased [29,30] and decreased [14] WG for GDM–F1 compared to controls was reported. In one study, the presence of GDM resulted in slower WG (based on weight for length) of GDM–F1 compared to non-GDM peers. The authors suggested that excess cord blood leptin and hormone levels of the intrauterine environment may have altered blood insulin levels, decreased appetite and increased metabolism during early infancy and, as such, may have contributed to the observed lower WG [14]. The study reporting a higher WG in GDM–F1 was conducted in infants born from women with both obesity and GDM versus lean women with NGT [30]. The co-existence of maternal hyperglycemia and high maternal BMI could potentially explain the observed increased WG among their offspring.

Given the impact of tempo of infant weight gain in the first few months of life on later life health outcomes [28], the observed alterations in the WG of GDM–F1 emphasize the importance of careful monitoring of GDM–F1 during early life.

### 2.3. Body Mass Index Trajectory in GDM–F1

BMI was the most commonly reported growth characteristic among offspring of GDM mothers. Sequential measurements of BMI enable the identification of the timing of adiposity rebound, the period when weight gain velocity increases due to a rise in the number and size of adipocytes; it has been suggested that early adiposity rebound is a marker for later obesity risk [31]. To envisage childhood obesity and to construct preventive strategies among GDM–F1, understanding longitudinal assessment of BMI, timing of adiposity rebound, and the subsequent risk of overweight/obesity (ow/ob) development are of importance [11].

Based on the reviewed studies which measured BMI (for at least two time points during the study), from birth till 5 y, no differences in BMI were observed between GDM–F1 and NGT–F1 [12,19,26,32,33]. There was one exception, a study which reported higher BMI at approximately 1 y in large for gestational age infants born to women with GDM (GDM-LGAF1) versus large for gestational age infants born to women with normal glycaemia (NGT–LGAF1) [17].

Studies with a longer time follow up indicated higher BMI values in infants of GDM women at a later age of 5 y [22], 7 y [12,18], around 10 y [34], 5–10 y [26], versus control groups, although others did not [19,35].

One study investigated and reported an earlier timing of adiposity rebound in GDM–F1 compared to NGT–F1 (4.4 y vs. 5.5 y) [24].

A few longitudinal studies examined BMI trajectories of two sub-groups of GDM–LGAF1 and GDM–nonLGAF1 beyond 1 y of age. Two studies suggested a higher BMI at 7 y [18], and BMI SDS at 8–14 y [11] for GDM–LGAF1 compared to GDM–nonLGAF1. The observed higher BMI trajectory of GDM–LGAF1 is in line with findings from cross sectional studies that GDM–LGAF1 at birth had increased fat mass and decreased lean mass compared to NGT–LGAF1 and suggests that their disproportionate body composition at birth could track in later life [36].

In line with the higher BMI values, 5 out of 10 studies focusing on the first 7 y of life showed 4–12% higher percentages of ow/ob in GDM–F1 compared to NGT–F1 or DM–F1 [12,13,19,24,37]. Likewise, in children with median age of 11 y, the HAPO study indicated that GDM–F1 had higher percentage of overweight or obesity compared to the NGT–F1 (29% vs. 39%) [38]. However, it must be noted that the use of different BMI cut offs and criteria, including International Obesity Task Force (ITOF), Centers for Disease Control (CDC), and country specific cut-offs make it difficult to compare the prevalence of overweight and obesity across studies.

Based on the available evidence it seems that, in addition to the effect of maternal GDM on short-term infant growth outcomes such as being born LGA or having a higher weight gain during infancy, maternal GDM may also have long-term adverse effects on BMI development trajectories during later life. Hence, it is crucial for parents and health care workers to consider this risk even when not being born big in GDM–F1 and, as such, to monitor BMI longitudinal patterns till late childhood/early adolescence [11].

### 2.4. Skin Fold Thickness Trajectory among GDM–F1

Assessing fat deposition in addition to BMI could clarify whether adiposity could be a plausible underlying mechanism related to increased metabolic risks reported for GDM–F1. Consistently higher SFTs were reported at birth in GDM–F1 versus NGT–F1 [17,33,34,37] (Table 1). These differences remained for the first months of life, although only investigated in a few studies [29,30], At 1 y of age the differences observed at birth and early life were no longer apparent [17,21,22,30], and limited information was available on SFT between 2–5 y of age.

Interestingly, at an older age (5–10 y), in line with the increased BMI values reported, the higher SFT in the GDM–F1 seen at birth re-appeared; it was consistently evident at 5 y [22], 7 y [18,37], around 8 y [35], and around 10 y [34]. Likewise, the HAPO follow up study confirmed higher SFT of GDM–F1 around 11 y old compared to NGT–F1 [38].

Taken together these findings reinforce the concept of Pedersen’s hypothesis, which postulates that increased maternal hyperglycemia, resulting in fetal hyperinsulinemia, may stimulate adipose tissue growth in infants [39]. The age-dependent disappearance and re-emergence of the association between maternal GDM and markers of adiposity in offspring suggests that GDM could have a differential impact on lean and fat mass development [40].

Interestingly, two studies evaluated fat mass distribution using either ultra-sonographic imaging [30] or body magnetic resonance imaging [29] in infants. Both studies indicated that visceral adiposity in GDM–F1 was amplified in infancy at a median age of 10 w [29] and at approximately 1 y [30]. Given the known tracking of adipose tissue depots throughout infancy and childhood, and their role in later childhood obesity [41,42], this illustrates the potential impact of the observed different pattern of fat mass distribution on later metabolic health among GDM–F1. In conclusion, there is a clear need for more longitudinal examinations of anthropometric and adiposity development, preferably with more frequent assessments in the first year of life and yearly assessments thereafter up to late childhood, as well as distribution of adiposity in GDM–F1 both in early infancy and childhood, in order to truly map and understand the potential long-term health implications.

### 2.5. The Relationship between Early Life Nutritional Modifiable Factors and Trajectory of Growth among GDM–F1

Offspring of GDM may provide us with a unique population to study and gain more insights into the origins of obesity. It is likely that alterations of adiposity among GDM–F1 are influenced by several pre- and post-natal factors. In this review, we specifically focused on the influence of the pre- and post-natal maternal diet as well as offspring milk feeding patterns on growth outcomes in GDM–F1. However, other pre-natal modifiable factors including maternal pre-pregnancy BMI and gestational weight gain may affect the association between maternal glucose tolerance and changes in growth outcome [9,11,14,22,34]. The impact of these pre-natal factors might even have an interaction with GDM; the combination of maternal GDM and obesity has a greater impact on offspring growth outcomes than the single factors alone [38]. Therefore, their independent effects as covariant have been examined in several of the included studies.

### 2.6. Pre- and Post-Natal Maternal Diet

Dietary control is normally the first line of treatment for women with GDM. It should be culturally appropriate, individualized and promote adequate and healthy nutrition for mothers and fetus [43]. Hence, nutritional advice tailored to an individual’s situation and phenotype will become a key asset to control maternal blood glucose [5].

Most of the modified dietary recommendations for women with GDM are from studies which only investigated the association of maternal diet and offspring growth and body composition at one time point—either at birth [44,45] or later in childhood [46]. For example, the recent systematic review by Yamamoto et al. concluded that dietary interventions for women diagnosed with GDM in RCT studies favorably affected birth outcomes, with lower birth weights (170 g), and less relative risk of macrosomia (0.49) [47]. Whereas, the DANISH birth cohort longitudinally assessed the intergenerational effect of maternal nutrition of women on repeated parameters of growth during infancy up to early or later childhood. The result showed that a high intake of refined grains (>4.3 servings/day vs. <1.8 servings/day) increased the risk of obesity at 7 y among GDM–F1 but no association was observed between high intake of grain and BMI z-score at 5 month and 1 y [48]. This study also showed that diets with low quality foods, for example low level of fiber content, may alter satiety hormone profiles and expression of genes involved in glucose and lipid metabolism and increase adiposity in the offspring.

Another subgroup analysis study from the Danish National Birth Cohort focused on the quality of carbohydrates in relation to longitudinal infant growth outcomes among GDM–F1. They demonstrated that consuming high glycemic index (GI) and glycemic load (GL) diets were related to a modest increase in the relative risk of LGA after adjustment and potential increase in total fat mass percentage (30% vs. 26%) and abdominal fat mass percentage (26.8% vs. 21.3%) among GDM–F1 vs. NGT–F1 at around 9–16 y [49].

In addition to the quality of the maternal diet, the timing of nutritional disturbances and insults during different trimesters may have specific physiological relevance on alterations in pancreatic structure or secretory capacity in offspring. Adipose and pancreatic tissue structural development starts already in first trimester, but fetal insulin secretion normally only starts during the second half of the pregnancy [49]. In order to assess the longitudinal and intergenerational impact of perinatal nutritional intervention among women with GDM on growth outcomes, it is crucial to focus on quality and quantity of diet, particularly carbohydrates, and timing of the nutritional intervention. In addition, early detection of hyperglycemic risk may be essential to prevent fetal growth deviations that result in LGA or macrosomic births.

### 2.7. Feeding Patterns (Including Breast Feeding (BF))

Breastmilk is the best and sole source of nutrition for infants in the first six months of life [50].

A recent review by Rzehak et al. showed that, in a general pediatric population, a shorter duration of BF (<3 m compared to ≥3 m) was associated with more rapid growth in early childhood and a higher risk of obesity in later in life [51]. The effect of breast feeding (BF) of women with GDM on WG and trajectory of BMI in GDM–F1 was assessed in only a couple of longitudinal studies [27,32,52]. In these studies, the impact of partial breastfeeding as well as full breastfeeding was evaluated, however the role of complementary feeding was not evaluated. However, it has been suggested that the influence of timely complementary feeding needs to be further investigated [53]. Adequate (full or partial) BF (>6 m) versus low (<6 m) decreased BMI among GDM–F1 with multi-ethnic backgrounds [52]. In addition, full or partial breastfeeding up to 5 m reduced BMI *Z*-score from 0–5 w (0.7 kg/m^2^) and 5 w–5 m (0.9 kg/m^2^) in the offspring of Danish women with GDM [27]. In contrast, full or partial breastfeeding (>4 m BF versus no BF) accelerated conditional WG (0.72 g) and BMI *Z*-score (0.49 kg/m^2^) only from 0–6 m in offspring of Asian women with GDM [32]. Hence, while any duration of BF has a positive effect on reduction of the risk of obesity in NGT–F1, based on these few studies, only a prolonged duration of BF seems to mitigate the risk imposed in utero for higher WG and BMI among GDM–F1 [53].

## 3. Conclusions

Longitudinal growth characteristics in GDM–F1 during early postnatal life and childhood are scarcely studied and the retrieved studies showed a large heterogenicity in design. However, our review still highlights the growing, but limited, number of reports showing deviations in BMI and SFT in of GDM–F1 compared to NGT–F1 and other defined control groups. GDM appears to have a time-specific effect on the quality of growth in the offspring marked by an indication for increased weight (gain) and adiposity (SFT) particularly at birth and increased adiposity (SFT and BMI) in later childhood (5–10 y). Interestingly, a limited evidence base suggests that the adverse health impact of exposure to maternal GDM on offspring adiposity and growth outcomes may be attenuated by improving healthy maternal diet and prolonged duration of BF.

## Figures and Tables

**Table 1:** Effect of exposure to gestational diabetes mellitus (GDM) on growth characteristics of offspring (compared to various control groups).

Category	Subcategory	Weight Gain (Exact Time Point)	Skinfold Thickness (Exact Time Point)	Body Mass Index (Exact Time Point)
**0–5 y**	0 (Birth)		Increased (birth) [17] ^b^ [22,33,37] ^c^	No difference (birth) [17,19] ^b^ [32,33] **^×^
	0–6 m	Decreased (0–6 m) [14] * Increased (0–6 m) [30] ^a^ Increased (11 d to 8–12 w) [29]	No difference (11 day) [29] Increased (6 w) [30] ^a×^ Increased (8–12 w) [29] No difference (4 m) [30] ^a×^	No difference (1–6 m) [19] ^×^
	0–1 y	No difference (0–1 y) [20,21] ^d^		No difference (6 m–1 y) [19]
	1 y		No difference (1 y) [30] ^a^ [17,21,22] ^c×^	No difference (1 y) [33] **^×^ [22] ^×^ Increased (1 y) [17] ^b^
	2 y		Increased (2 y) [22] only girl ^×^	No difference (2 y) [33] **^×^
	3 y			No difference (3 y) [33] ** [26] ^d^ [12]
	4 y			No difference (4 y) [26] ^d^ [12] ^×^
	2–5 y			No difference (2–5 y) [19] ^×^
**5–10 y**	5 y		Increased (5 y) [22] ^c×^	Increased (5 y) [22] only girl ^×^
	≥ 7 y		Increased (7 y) [18] ^b×^ [37] (only girl)	Increased (7 y) [18] ^b×^ [12] **^×^
			Increased (7.1 –8.7 y) [35] ^fj^	No difference (7.1–8.7 y) [35] **^j×^
	5–10 y		Increased (9. 5 y) [34] c (only girl)	Increased (9.5 y) [34] (only girl)
				Increased (5–9 y) [26] ^d×^
				No difference (5–10 y) [19] **
**≥ 10 y**			Increased (median 11 y) [38]	Increased (10–14 y) [11] ^b×^ No difference (14–17 y) [16] ^e×^

* WFL Z = weight for length Z score; ** BMI Z = body mass index Z score; **^×^** Longitudinal studies measured trajectory of body mass index or skin fold thickness at more than two time points; ^a^. Ob–GDM–F1 vs. (Lean–NGT–F1 or Ob–NGT–F1) = offspring born to mother with high pre–pregnancy BMI and gestational diabetes vs. mother with normal BMI and normal blood glucose tolerance or mother with high pre-pregnancy BMI and normal blood glucose; ^b^. GDM–LGAF1 vs. NGT–LGAF1 = large for gestational age offspring born to mothers with gestational diabetes vs. mothers with normal glucose tolerance; ^c^. Subscapular and tricepses; ^d^. GDM–F1 vs. IGT–F1= offspring born to mothers with gestational diabetes vs. mothers with intolerance glucose test; ^e^. GDM–F1 vs. T1DM + T2DM = offspring born to mothers with gestational diabetes vs. mothers with type one or two diabetes; ^f^. Subscapular and triceps; ^j^. GDM– vs. IGT vs. IH vs. NGT = offspring born to mothers with gestational diabetes vs. intolerance glucose test vs. isolated vs. normal glucose tolerance.

## Supplementary Materials

The following are available online at https://www.mdpi.com/1660-4601/17/9/3187/s1. Table S1: Study characteristics of the reviewed articles measured weight, weight gain, body mass index or skin fold thickness (at least at two time points); Table S2: Timepoints of anthropometric measurements; Supplementary Information: Study selection for qualitative review.
