# Supplementary material for: Gestational Diabetes Mellitus Is Associated with Age-Specific Alterations in Markers of Adiposity in Offspring: A Narrative Review
Source: Int J Environ Res Public Health. 2020 May 4;17(9):3187. doi: 10.3390/ijerph17093187 (PMC7246521; doi:10.3390/ijerph17093187)
Supplement: Supplementary file 1 [file ijerph-17-03187-s001.zip › Supplementary Method Information.docx]

Supplementary Information: Study selection for qualitative review

The publications evaluated to be reviewed in our paper were published between 1995 – 2019 (August) and retrieved using the following search string: (pregnant OR pregnancy) AND (GDM OR "gestational diabetes" OR "gestational hyperglycemia" OR "gestational hyperglycaemia" OR "hyperglycaemia during pregnancy" OR “hyperglycaemia during pregnancy" OR "pregnancy glycemia" OR "gestational glycemia" OR "gestational glycaemia" OR " pregnancy glycaemia") AND (child OR children OR offspring OR childhood) AND (growth OR "growth trajectories" OR anthropometric OR "high BMI" OR "high body mass index“ OR "childhood obesity" OR BMI OR "obesity in offspring" OR "longitudinal trajectory of growth" OR "longitudinal growth trajectory" OR "offspring adiposity").

Additionally, the search results were imported into EndNote and articles evaluated in two steps; initial title and abstract screening followed by full text screening of those that passed the initial screening. Studies were included for review if they met all the following defined inclusion criteria: 1) written in English; 2) clinical data; 3) original research paper; 4) mothers were diagnosed with GDM, regardless of diagnostic criteria used and without comorbid type one or type two diabetes and 5) at least one growth parameter (weight, BMI, SFT) of offspring born to mother with GDM (GDM–F1) measured at two different time points.

Following our inclusion criteria, we retrieved a total of 25 studies in which the growth outcomes of GDM–F1 offspring was compared to those of infants born to mothers with normal glucose tolerance (NGT–F1), type 1 or type 2 diabetes. In addition, two of these publications provided more details comparing the growth outcomes of large for gestational age infants born to women with GDM (GDM–LGAF1) vs appropriate for gestational age infants (GDM–AGAF1). Lastly, among the retrieved studies we focused on studies that also provided information on the role of maternal diet (2 studies) and breastfeeding (3 studies) on longitudinal growth outcomes of GDM–F1.
