# Supplementary material for: Gestational Diabetes Mellitus Is Associated with Age-Specific Alterations in Markers of Adiposity in Offspring: A Narrative Review
Source: Int J Environ Res Public Health. 2020 May 4;17(9):3187. doi: 10.3390/ijerph17093187 (PMC7246521; doi:10.3390/ijerph17093187)
Supplement: Supplementary file 1 [file ijerph-17-03187-s001.zip › Table 1 and 2 Supplements-revised.docx]

| Supplementary Table 1. Study characteristics of the reviewed articles measured weight, weight gain, body mass index or skin fold thickness (at least at two time points) | | | | | | | | | | | |
| --- | --- | --- | --- | --- | --- | --- | --- | --- | --- | --- | --- |
| **First author, Year** | **Country** | **Type of study** | **Mother–offspring number** | **Ethnicity** | **GDM diagnostic criteria** | **Gestational week at OGTT** | **GDM–F1 vs other group= × ; GDM–F1 subgroup= ××** | **W** | **WG** | **BMI** | **SFT** |
| Aris, 2017 | Singapore | Prospective | GDM–F1 = 181 NGT–F1 = 835 | Chinese Malay Indian | WHO^1^ | 26 – 28 w | **× ××** | √√* | √√* | √√* |  |
| Baptiste- Robert^⁑^, 2012 | US | Prospective | GDM–F1 = 484 NGT–F1 = 27,874 | Caucasian African–American | NCP ^2^ |  | **×** | √√ |  | √√* |  |
| Borgono, 2012 | Canada | Prospective | GDM–F1 = 36 NGT–F1 = 68 | Caucasian Other | NDDG^3^ | late 2^nd^ trimester | **×** |  | √^ |  |  |
| Fenger–Grøn, 2015 | Denmark | Prospective | GDM–F1 = 131 WHO ref/ Danish ref | Caucasian | Danish criteria ^4^ |  | **×** | √√ |  | √√ |  |
| Hamilton, 2010 | Canada | Prospective | GDM–F1 = 76  IGT–F1 = 46 NGT–F1 = 150 | Caucasian Asian African–American  Other | NDDG ^3^ | 27 – 33 w | **×** | √√ | √ |  | √ |
| [Hammoud](https://www.ncbi.nlm.nih.gov/pubmed/?term=Hammoud%20NM%5BAuthor%5D&cauthor=true&cauthor_uid=29492638), 2018 | Netherland | Prospective | GDM–LGAF1 = 24 GDM–AGAF1 = 80 NGT Dutch ref | Caucasian Non–Caucasian | ND | ND | **× ××** | √√ |  | √√ |  |
| Hakanen^⁑^, 2016 | Finland | Prospective | GDM–F1 + T1DM–F1 + NGT–F1 = 6906 | ND | Finland criteria ^5^ | 12 – 14 or  24 – 28 w | **×** |  |  | √√ |  |
| Hillier^⁑^, 2016 | US | ND | NGT–F1 = 10,647 GDM–F1 = 707 | Hawaiian Caucasian Non–Caucasian | C & C^6^ | ND | **×** | √√ |  | √√ |  |
| Kramer, 2014 | Canada | Prospective | GDM–F1 = 90 NGT–F1 = 250 | Caucasian Asian Other | NDDG ^3^ | Median 30w | **×** | √√ |  |  |  |
| Krishnaveni, 2005 | India | Prospective | GDM–F1 = 41  NGT–F1 = 548 | Indian | C & C^6^ | 28 – 32 w | **×** | √√ |  | √√ | √√^^ |
| Krishnaveni, 2010 | India | Prospective FU study | GDM–F1 = 35 NGT–F1 = 440 | Indian | C & C^6^ | 28 – 32 w | **×** |  |  | √√ | √√^^ |
| Lee, 2007 | Korea | Prospective | GDM–F1 = 202 IGT–F1 = 96 | Asian | NDDG ^3^ | 28 – 32 w | **×** | √ |  | √√* |  |
| Logan, 2016 | UK | Prospective | GDM–F1 = 42 NGT–F1 = 44 | Caucasian  African–American Asian Mixed | UK criteria ^7^ | 26 w  30 w | **×** | √√ | √ |  | √√֍ |
| Megia, 2015 | Spain | Prospective | GDM–F1 = 79  NGT–F1 = 79 | Caucasian | NDDG ^3^ | 26 – 30 w | **×** | √ |  | √√* | √ |
| Parker, 2011 | US | Prospective | GDM–F1 = 35  NGT–F1 = 621 | ND | ADA ^8^ | 26 – 28 w | **×** |  | √ |  |  |
| Pirkola, 2008 | Finland | Prospective | GDM–F1 = 22 T1DM–F1 = 16  NGT–F1 = 25 | Caucasian | Finland criteria ^5^ | 26 – 28 w | **×** | √√* |  |  |  |
| Pham^⁑^, 2013 | US | Retrospective | GDM–F1 = 255  NGT–F1 = 1,838 | Caucasian  African–American Latina Asian | C & C ^6^ | 24 – 28 w | **×** | √√ |  |  |  |
| Regnault, 2013 | US | Prospective | GDM–F1 = 43 IGT–F1 = 32 IH–F1 = 87 NGT–F1 = 796 | African–American  Hispanic Other | ADA ^8^ | 26 – 28 w | **×** | √√ |  | √* | √^^ |
| Silverman, 1998 | US | Prospective | GDM–F1 = ND NGT–F1 = ND T1DM+T2DM = ND | Caucasian African–American Hispanic Other | ND | ND | **×** |  |  | √√ |  |
| Tam ^⁑^, 2017 | Hong Kong | Prospective | GDM–F1 = 131 NGT–F1 = 794 | Chinese | WHO^1^ | 24 – 32 | **×** | √√ |  |  | √√ |
| Uebel, 2014 | Germany | Prospective | Ob–GDM–F1 = 16  Ob–NGT–F1 = 13 Lean–NGT–F1 = 15 | Caucasian | HAPO ^9^ | 2^nd^ and 3^rd^ trimesters | **×** | √√ | √ |  | √√ |
| Vohr, 1997 | US | Prospective | GDM–LGAF1 = 47 GDM–AGAF1 = 47  NGT–LGAF1 = 55 NGT–AGAF1 = 44 | Caucasian African–American Hispanic | C & C ^6^ | 24 – 28 w | **×** **××** | √√ |  | √√ | √√ |
| Vohr, 1999 | US | Prospective FU study | GDM–LGAF1 = 47 GDM–AGAF1 = 59  NGT–LGAF1 = 46 NGT–AGAF1 = 55 | Caucasian African–American  Hispanic  Asian | C & C ^6^ | 24 – 28 w | **×** **××** | √√ |  | √√ | √ |
| Whitaker^⁑^, 1998 | US | Retrospective | DT–GDM–F1 = 58  NGT–F1 = 257 | Non–Hispanic Caucasian | C & C ^6^ | 24 – 32 w | **×** |  |  | √√* |  |
| Crume, 2012 | US | Retrospective | Adequate BF–GDM = 41 Low BF–GDM = 53 | Hispanic Non– Hispanic African–American | NDDG ^3^ | 24 – 28 w | **××** | √ |  | √√ |  |
| Leng ^⁑^, 2015 | China | Retrospective | GDM–F1^∞^= 1263 | Chinese | China study criteria ^10^ | 26 – 30 w | **××** | √√* | √ | √ |  |
| Lowe, 2018 | HAPO cites | Prospective FU study | GDM–F1 = 672 NGT–F1 = 4025 | Non-Hispanic Hispanic Asian Others | IADPSG ^11^ | 28 w | **×** | √√ |  | √ | √ |
| Maslova, 2018 | Denmark | Prospective | GDM–F1= 608 | Caucasian | Danish criteria ^4^ |  | **××** | √√ |  | √√ |  |
| Zhu ^⁑^, 2016 | Denmark | Prospective | GDM–F1 = 661 | ND | WHO^1^ or Danish criteria ^4^ | 30 w | **××** |  |  | √√* |  |
| Zhu, 2017 | Denmark | Prospective | GDM–F1 = 918 | ND | WHO^1^ or Danish criteria ^4^ | 30 w | **××** | √ |  | √√* |  |

√ = measured at one time point; √√ = measured more than one time point; ND = not define, W = weight, WG = weight gain, BMI= body mass index, SSFT = sum of skin fold thickness; LGAF1 = large for gestational age infants; AGA = appropriate for gestational age infants; FU study = follow up study; GDM–F1 = offspring born to women with Gestational diabetes; NGT–F1 = offspring born to women with normal glucose tolerance; T1DM = type 1 diabetes mellitus, T2DM = type 2 diabetes mellitus * Z score; ⁑ Percentage of ow/ob = overweight/obesity, ^WG reported as WFL Z =weight for length Z-score; ^^SFT reported as subscapular and triceps skinfold; ^∞^ number based on BMI category: group A <24 kg/m^2^ = 823, group B 24 –27.9 kg/m^2^ = 335, group C ≥ 28 kg/m^2^ = 105, and gestational weight gain: group D ( inadequate) = 156, group E (adequate )= 394, group F (excessive) = 713 ; ֍ total adipose tissue and changes in total adipose tissue (AT) cm^3^; Internal abdominal AT/ subcutaneous AT ratio by magnetic resonance imaging (MRI).**1**. **WHO** **= Word Health Organisation** as 2 h 75 g oral glucose tolerance test (OGTT) and gestational diabetes (GDM) diagnosed if glucose threshold for fasting ≥ 7.0 or 2 h > 7.8 mmol/L; **2. NCP** = National Collaborative Perinatal as 3 h 100 g OGTT and GDM diagnosed if glucose threshold for fasting ≥ 5.8, 1 h ≥ 10.6, 2 h ≥ 9.2 or 3 h ≥ 8.1 mmol/L; **3. NDDG = National Diabetes Data Group** as two steps; the first step 50 g glucose challenge test (GCT) and glucose threshold for 1 h ≥ 7.2 mmol/L then second step as 3 h 100 g OGTT and (i) GDM diagnosed if glucose threshold for fasting ≥ 5.8 , 1 h ≥ 10.6, 2 h ≥ 9.2, or 3 h ≥8.1 mmol/L and (ii) gestational impaired glucose tolerance (GIGT) diagnosed if glucose threshold for only one of the cut off; **4. Danish criteria** as 2 h 75 g OGTT and GDM diagnosed if glucose threshold for 2 h ≥ 9.0 mmol/L; **5.** **Finland national guidelines** as 2 h 75g OGTT and GDM diagnosed if glucose threshold for one or more values above, fasting ≥ 4.8, 1 h ≥ 10.0 or 2 h ≥ 8.7 mmol/L**; 6. C & C = Carpenter and Coustan:** the first step 50 g GCT and glucose threshold for 1 h ≥ 7.8 mmol/L, then second step OGTT as 3 h 100 OGTT and GDM diagnosed if glucose threshold as two or more values above, fasting ≥ 5.3, 1 h ≥ 10.0, 2 h ≥ 8.7, 3 h ≥ 7.8 mmol/L; **7. United Kingdome criteria**, as two steps and the first step 50 g GCT and glucose threshold for 1 h > 7.8 mmol/L and second step OGTT and GDM diagnosed if glucose threshold for fasting ≥ 5.3 or 2 h ≥ 7.8 mmol/L; **8. ADA =** American Diabetes Association as two steps: the first step 50 g GCT and glucose threshold for 1 h ≥ 7.8 mmol/L and second step 3 h, 100 g OGTT and GDM diagnosed if glucose threshold for at least two values fasting ≥ 5.3, 1 h ≥ 10.0, 2 h ≥ 8.6, 3 h ≥ 7.8 mmol/L, and impaired glucose tolerance (IGT) as one abnormal glucose value on the OGTT, isolated hyperglycemia (IH) as abnormal GCT; **9.** **HAPO = Hyperglycemia and Pregnancy Outcome** as 2 h 75g OGTT and GDM diagnosed if at least one value above, fasting ≥ 5.1, 1 h ≥ 10, 2 h ≥ 8.5 mmol/L; **10. China study Criteria** either diabetes fasting glucose ≥ 7.8 or < 11.1 mmol/L or 2 h ≥ 11.1 mmol/L or IGT with 2 h ≥ 7.8 and <11.1 mmol/L) were regarded as GDM; **11.** **IADPSG** = International Association of Diabetes and Pregnancy Study Groups as 3 h 75 g OGTT if one value above fasting ≥ 92mg/dL; 1 h ≥ 180 mg/dL; 2 h ≥ 153 mg/dL.

| **First author, Year** | **Timepoints of anthropometric measurements (months)** | | | | | | | | | | | | | | | | | | | | | | | | | | | | | | | |
| --- | --- | --- | --- | --- | --- | --- | --- | --- | --- | --- | --- | --- | --- | --- | --- | --- | --- | --- | --- | --- | --- | --- | --- | --- | --- | --- | --- | --- | --- | --- | --- | --- |
|  | birth | 1 | | 2 | 3 | 4 | 5 | 6 | 9 | 11 | 12 | 14 | 15 | 18 | 24 | 26 | 36 | 45 | 48 | 60 | 66 | 72 | 84 | 96 | 108 | 120 | 132 | 144 | 156 | 168 | 180 | 192 |
| Aris, 2017† | x | |  |  | x |  |  | x | x |  | x |  | x | x | x |  | x |  |  |  |  |  |  |  |  |  |  |  |  |  |  |  |
| Baptiste-Roberts, 2012 | x | |  |  |  |  |  |  |  |  |  |  |  |  |  |  | x |  | x |  |  |  | x |  |  |  |  |  |  |  |  |  |
| Borgono, 2012 | x | |  |  |  |  |  |  |  |  | x |  |  |  |  |  |  |  |  |  |  |  |  |  |  |  |  |  |  |  |  |  |
| Hamilton, 2010 | x | |  |  |  |  |  |  |  |  | x |  |  |  |  |  |  |  |  |  |  |  |  |  |  |  |  |  |  |  |  |  |
| Hammoud, 2018† | x | | x |  |  | x |  | x | x | x |  | x |  | x | x |  | x | x |  |  | x |  |  |  |  |  | x |  | x |  |  |  |
| Hakanen, 2016^●^ | x | |  |  |  |  |  | x |  |  | x |  |  |  | x |  |  |  |  | x |  |  | x |  |  |  |  | x |  |  |  |  |
| Hillier, 2016^●^ |  | |  |  |  |  |  |  |  |  |  |  |  |  | x |  |  |  |  |  |  |  |  |  |  | x |  |  |  |  |  |  |
| Kramer, 2014 | x | |  |  | x |  |  |  |  |  |  |  |  |  |  |  |  |  |  |  |  |  |  |  |  |  |  |  |  |  |  |  |
| Krishnaveni, 2005 | x | |  |  |  |  |  |  |  |  | x |  |  |  | x |  | x |  | x | x |  |  |  |  |  |  |  |  |  |  |  |  |
| Krishnaveni, 2010 |  | |  |  |  |  |  |  |  |  |  |  |  |  |  |  |  |  |  | x |  |  |  |  | x |  |  |  |  |  |  |  |
| Lee, 2007 | x | |  |  |  |  |  |  |  |  |  |  |  |  |  |  | x |  | x | x |  |  |  |  |  |  |  |  |  |  |  |  |
| Logan, 2016 | x | |  | x |  |  |  |  |  |  |  |  |  |  |  |  |  |  |  |  |  |  |  |  |  |  |  |  |  |  |  |  |
| Lowe, 2018≈ |  | |  |  |  |  |  |  |  |  |  |  |  |  |  |  |  |  |  |  |  |  |  |  |  |  | x |  |  |  |  |  |
| Megia, 2015 | x | |  |  |  |  |  |  |  |  | x |  |  |  | x |  |  |  | x |  |  |  |  |  |  |  |  |  |  |  |  |  |
| Parker, 2011 | x | |  |  |  |  |  | x |  |  |  |  |  |  |  |  |  |  |  |  |  |  |  |  |  |  |  |  |  |  |  |  |
| Pirkola, 2008 | x | |  |  |  |  |  |  |  |  |  |  |  |  |  |  |  |  |  | x |  |  |  |  |  |  |  |  |  |  |  |  |
| Pham, 2013 | x | |  |  |  |  |  |  |  |  |  |  |  |  |  |  | x |  |  |  |  |  |  |  |  |  |  |  |  |  |  |  |
| Regnault, 2013† | x | |  |  |  |  |  | x |  |  |  |  |  |  |  |  | x |  |  |  |  |  | x |  |  |  |  |  |  |  |  |  |
| Silverman, 1998† | x | |  |  |  |  |  | x |  |  | x |  |  |  | x |  | x |  | x | x |  | x | x | x | x | x | x | x | x | x | x | x |
| Tam, 2017 | x | |  |  |  |  |  |  |  |  |  |  |  |  |  |  |  |  |  |  |  |  | x |  |  |  |  |  |  |  |  |  |
| Uebel, 2014 | x | | x |  |  | x |  |  |  |  | x |  |  |  |  |  |  |  |  |  |  |  |  |  |  |  |  |  |  |  |  |  |
| Vohr, 1997 | x | |  |  |  |  |  |  |  |  | x |  |  |  |  |  |  |  |  |  |  |  |  |  |  |  |  |  |  |  |  |  |
| Vohr, 1999 | x | |  |  |  |  |  |  |  |  |  |  |  |  |  |  |  |  | x | x |  | x | x |  |  |  |  |  |  |  |  |  |
| Whitaker, 1998* | x | |  |  |  |  |  | x |  |  | x |  |  |  | x |  |  |  |  | x |  |  |  |  |  | x |  |  |  |  |  |  |

Supplementary Table 2: Summary of timepoints of anthropometric measurements

†Statistical analysis did not report for all measurement timepoints; ^●^BMI repeated measurements but statistical analysis was available only for overweight/obesity or adiposity rebounding; *average age of measurements provided; ≈Follow up study.
